# A pachyderm perfume: odour encodes identity and group membership in African elephants

**DOI:** 10.1038/s41598-022-20920-2

**Published:** 2022-10-06

**Authors:** Katharina E. M. von Dürckheim, Louwrens C. Hoffman, Carlos Poblete-Echeverría, Jacqueline M. Bishop, Thomas E. Goodwin, Bruce A. Schulte, Alison Leslie

**Affiliations:** 1 Department of Conservation Ecology and Entomology, Faculty of AgriSciences, University of Stellenbosch, Stellenbosch, South Africa; 2 Department of Animal Sciences, Faculty of AgriSciences, University of Stellenbosch, Stellenbosch, South Africa; 3 Centre for Nutrition and Food Sciences, Queensland Alliance for Agriculture and Food Innovation (QAAFI), The University of Queensland, Digital Agricultural Building, 8115, Office 110, Gatton, QLD 4343 Australia; 4 Department of Viticulture and Oenology, Faculty of AgriSciences, University of Stellenbosch, Stellenbosch, South Africa; 5 Institute for Communities and Wildlife in Africa, Department of Biological Sciences, University of Cape Town, Private Bag X3, Cape Town, 7701 South Africa; 6 Department of Chemistry, Hendrix College, Conway, AR 72032 USA; 7 Department of Biology, Western Kentucky University, 1906 College Heights Blvd., #11080, Bowling Green, KY 42101-1080 USA

**Keywords:** Chemical ecology, Molecular ecology

## Abstract

Group-living animals that live in complex social systems require effective modes of communication to maintain social cohesion, and several acoustic, olfactory and visual signaling systems have been described. Individuals need to discriminate between in- and out-group odour to both avoid inbreeding and to identify recipients for reciprocal behaviour. The presence of a unique group odour, identified in several social mammals, is a proposed mechanism whereby conspecifics can distinguish group from non-group members. African elephants (*Loxodonta africana*) live in stable, socially complex, multi-female, fission–fusion groups, characterized by female philopatry, male dispersal and linear dominance hierarchies. Elephant social behaviour suggests that individuals use odour to monitor the sex, reproductive status, location, health, identity and social status of conspecifics. To date, it is not clear what fixed or variable information is contained in African elephant secretions, and whether odour encodes kinship or group membership information. Here we use SPME GC–MS generated semiochemical profiles for temporal, buccal and genital secretions for 113 wild African elephants and test their relationship with measures of genetic relatedness. Our results reveal the existence of individual identity odour profiles in African elephants as well as a signature for age encoded in temporal gland and buccal secretions. Olfactory signatures for genetic relatedness were found in labial secretions of adult sisters. While group odour was not correlated with group genetic relatedness, our analysis identified “group membership” as a significant factor explaining chemical differences between social groups. Saturated and short-chain fatty acids (SCFAs), derived from key volatile compounds from bacterial fermentation, were identified in temporal, buccal and genital secretions suggesting that group odour in African elephants may be the result of bacterial elements of the gut microbiome. The frequent affiliative behavior of African elephants is posited as a likely mechanism for bacterial transmission. Our findings favour flexible group-specific bacterial odours, which have already been proposed for other social mammals and present a useful form of olfactory communication that promotes bond group cohesion among non-relatives in fission–fusion mammals.

## Introduction

Olfactory communication is arguably the most important mode of communication in mammals, functioning in signals related to reproduction, mate choice and attraction, territoriality, parental care, kin discrimination and disease transmission, all of which inform population dynamics and structure^1,2^. Olfactory cues encode fixed and variable information such as individual identity^3,4^, age, rank, fertility^5^ and sex^6,7^, as well as genetic differences between individuals, populations, and species^8–10^. Research also reveals that olfactory cues may encode information about individual genetic quality and relatedness^11–13^, and provide geographic-specific information^14^. In doing so, olfactory cues may act to promote outbreeding, facilitate nepotism, and function in phenotype matching, kin discrimination and mate choice; and several studies have also explored the correlation between olfactory phenotype and genotype in these contexts^15,16^.

A number of acoustic, olfactory and visual cue systems have been described in group-living mammals where socially complex systems require effective means of communication to maintain social cohesion. Within a group context, individuals discriminate foreign conspecifics from group members to both avoid inbreeding and to identify recipients for reciprocal behaviour. Such olfactory group/herd/clan/colony identity signals have been described in many mammals including beavers^17^, bats^18^, meerkats^19^, naked mole rats^20^, chimpanzees^21^ and rhesus macaques^22^. Measures of group odour have been correlated to genetic relatedness^23^, as well as to chemical differences in microbiomes and bacterial metabolic by-products^24,25^. Among elephants, the use of olfactory cues are central to communication. African (*Loxodonta africana*) and Asian elephants (*Elephas maximus*) of both sexes engage in distinctive trunk-tip behaviours when inspecting conspecific genitalia, temporal glands, mouth, ears and feet^26^. Elephants emit chemical signals via their temporal glands, urine, faeces, breath, saliva, interdigital glands, genitalia and body surfaces, and detect both self and non-self signals via highly sophisticated olfactory and vomeronasal systems^27^. These chemical signals influence elephant behavior, social interactions and reproduction^28^. Elephant reliance on olfaction is also reflected in its genome, comprising ∼2000 functional olfactory receptor genes and > 2200 pseudogenes, the highest number of olfactory receptor genes of any mammal^29^.

To date, chemosensory research in elephants has primarily focused on captive elephants and the role of sexual signaling in captive bulls in musth^30–33^ Elephants have a distinctive temporal gland, a modified apocrine structure imbedded in the subcutaneous tissue on each side of the head between the eye and the ear. Elephant bulls greet one another by reaching the trunk to a same sex conspecific’s mouth, temporal gland, or genital region. The semiochemistry of temporal gland secretions is consequently relatively well studied in male elephants^34^, but little attention has been paid to females. Female mammals also use scent signaling for sexual attraction, mediation of female competition, cooperation and to facilitate maternal behaviour^6,17,35,36^. Unlike adult Asian elephant females, African elephant adult females secrete frequently from the temporal gland, particularly when separated groups reunite, and when distressed^37^. When related females and their offspring meet one another, elephants perform an elaborate greeting ceremony, in which elephants rub their bodies against one another, trumpet, rumble, entwine their trunks, click tusks, emit temporal gland secretions (TGS), and release urine and dung profusely, while fanning their uplifted ears and spinning their bodies^37^. Although no empirical studies have explained the function and the content of these chemical emissions in African elephant females, it is likely that these odour signatures underlie recognition in elephants and possibly promote bond group cohesion^38,39^.

Despite evidence for the importance of olfaction in African elephant communication and behaviour^40–42^, research on the semiochemistry in African elephants is limited due to the challenges and dangers of obtaining high quality odour samples from a wild population. Unlike Asian elephant society where dominance hierarchies are weak and non-linear due to divergent resource dynamics^43^, African elephant social groups are characterized by female linear dominance hierarchies defined by age and size^44^. Males leave their natal family at approximately 14 years of age, often joining all-male coalitions led by older bulls^45^. Social stability and dominance in all-bull groups is regulated by reproductive and dominance states termed musth, which is chemically encoded in TGS of bulls^34^ and mediated by microbes^46^. Female African elephants are philopatric, living in multi-tiered, matrilocal, fission–fusion groups comprised of adult females and their offspring^39,47^. Within populations, ‘core’ groups, often called ‘families’, are composed of predictable sets of individuals, containing 1–20 adult females and their immature offspring^44^. Families can divide into units as small as a single adult female and her immature offspring, or families can fuse with other families to form bond groups or larger aggregations. Associations between adult females are enduring, with fission–fusion dynamics determined by ecological and genetic factors. Genetic relatedness among elephants is a determining factor driving core group formation among first-order maternal relatives^44^ suggesting that the evolution of cooperative social behaviour enhances inclusive fitness through the effects of genetic structuring. Yet, among elephant populations highly depredated by poaching, genetic relatedness was not found to be a limiting factor regulating sociality^47^. In combination, results suggest that genetic relatedness is important to core group formation among adult females, and that factors such as prior familiarity and social status may further contribute towards social structuring. Inter-individual recognition among kin and socially preferred conspecifics has important fitness consequences for elephant survival and studies on auditory communication suggests that African elephants discriminate among kin, familiar and socially affiliated individuals using acoustic cues^48,49^.

Generally African elephant males avoid breeding with kin, however the mechanism by which bulls recognize kin is hitherto unknown^50^. In female African elephants distinguishing individual olfactory cues could be relevant where kinship and dominance rank assessments underpin hierarchical structuring and fission–fusion dynamics and mediate cooperative and competitive interactions for example through cooperative offspring care and group defense^39^. Natural selection could favour group-specific odours that promote bond group cohesion and increase fitness benefits for example cooperative offspring care and cooperative group defense. The aim of this study therefore was to analyse the semiochemistry of buccal, genital and temporal gland secretions to determine whether body odour in wild African elephants conveys information about genetic relatedness and social relationships. We predicted that (1) genetic relatedness would be significantly correlated with odour in family units, (2) adult sisters have more similar TGS odour profiles than unrelated adult females, and (3) temporal gland, buccal and genital chemical profile encode elephant attributes such as age and sex. Lastly, we provide here an insight into the fixed and variable information contained in elephant chemical profiles.

## Results

### Sample population

DNA and temporal, buccal and genital swabs were obtained from 113 elephants translocated in 15 family groups in Majete Wildlife Reserve, Malawi (see Table S1 in Supplementary Information). Subjects included 40 adult females (of which 39 were estimated to be over the age of 25), one adult male, 9 sub-adults, 32 juveniles, 19 calves and 12 infants (Table 1). Females were classified as adult if they were reproductively active (over the age of 15). Park management targeted family units for translocation, hence the absence of mature males.

**Table 1:** Age/sex classes of sampled elephants from the translocated population.

Age category	Age class	Males	Females	Total per age class
Infants I	0–1	7	5	12
Calves II	2–4	14	5	19
Juveniles III	5–9	15	17	32
Sub-adults IV	10–14	0	9	9
Adults V	15 +	1	40	41
Total		37	76	113

### Chemical data

The removal of substances from blanks or those present in only one sample reduced the number of peaks present in each sample significantly. For most samples, only minor if any shifts, were required. Lastly, the retention times of homologous peaks in the aligned dataset were left-skewed, suggesting that the variation among most substances was less than 0.05 min. Temporal samples were collected for analysis from 106 elephants, and 169 temporal substances retained after all filtering steps. Genital samples were collected from 109 elephants, 308 genital substances retained after all filtering steps. Buccal samples were collected from 106 elephants, and 370 buccal substances retained after all filtering steps. Only compounds soluble in GC–MS were extracted and some substances may have further metabolized post sampling.

### Comparisons of putative chemical compounds derived from buccal, labial and temporal gland secretions of adult females

**Figure 1:**
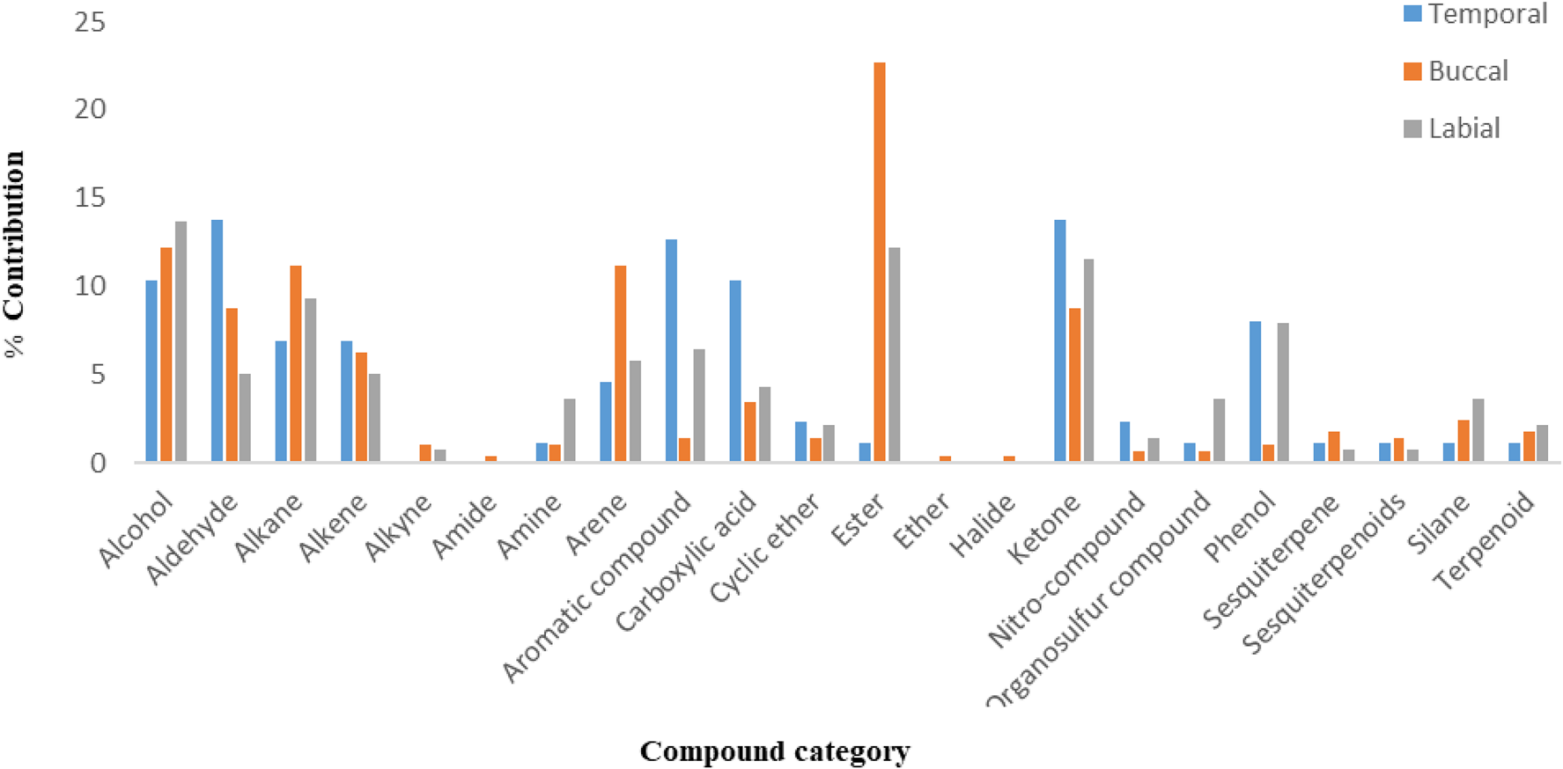
Major chemical compound categories (%) in temporal, buccal and labial secretions in African elephants.

We detected mostly aldehydes, ketones, aromatic compounds and carboxylic acids in TGS, followed by alcohols and phenols (Fig. 1). TGS contained significantly more aromatic compounds and carboxylic acids than breath or labial secretions. Labial secretions comprised mostly alcohols, esters, and ketones, followed by alkanes and phenols. Buccal secretions contained significantly more esters than TGS or labial secretions, but had fewer ketones and phenols than TGS or labial secretions^51^.

### Genetic data

**Figure 2:**
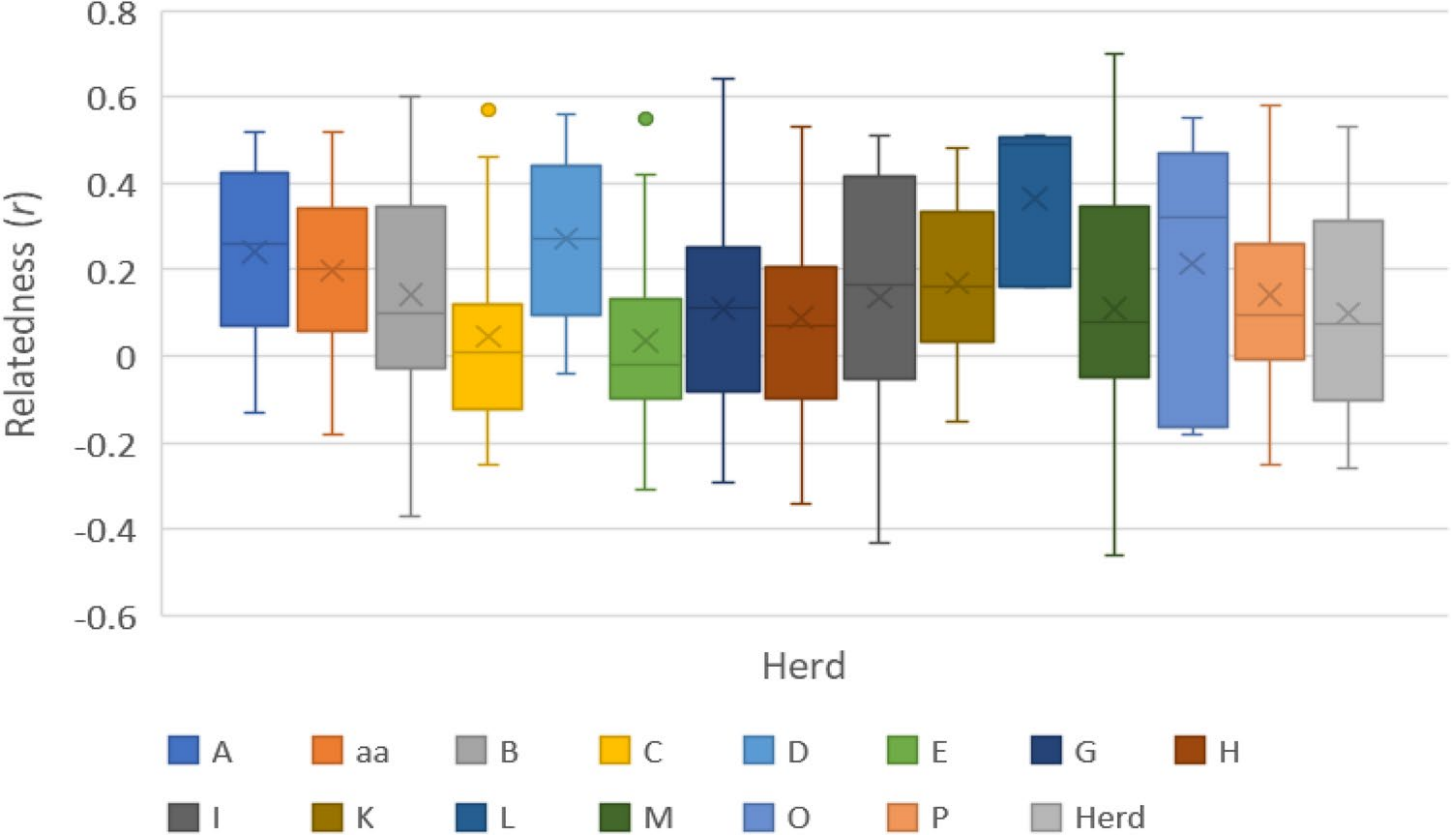
Pairwise relatedness estimates (*r*) per family group (herd).

DNA samples were genotyped at 18 highly polymorphic microsatellite loci (~ no alleles/locus = 5.8, proportion of loci typed = 0.99 and expected heterozygosity = 0.65). No loci deviated significantly from the Hardy–Weinberg Equilibrium (HWE) and were therefore all retained for subsequent analyses. Pairwise estimators for relatedness followed a normal distribution. In the translocated population, groups showed mean relatedness coefficients within the range of their expected distribution (related maternal lineage and their offspring together with unrelated individuals), ranging from a minimum QG*r* of −0.46 to a maximum of 0.7 (Fig. 2). Mother–offspring pairs that had maternity match probabilities of 100% were retained for further analysis.

### Chemical similarity (ANOSIM)

Using whole profile comparisons an analysis of similarity (ANOSIM) based on Bray–Curtis indices was used to test for an effect of sex, age cohort and group membership cohorts).

#### Age and sex differences

Significant differences in odour profiles were found between age categories in temporal gland (ANOSIM, global *R* = 0.26, p < 0.031) and buccal secretions (ANOSIM, global *R* = 0.39, p < 0.0001). An effect tending towards significance could be found for sex in temporal gland secretions (ANOSIM, global *R* = 1.27, p < 0.07) and genital secretions (ANOSIM, global *R* = 0.14, p < 0.07).

#### Group differences

Multivariate statistical analyses of the relative proportion of each substance sampled from the 15 family groups revealed highly significant differences in chemical profiles of temporal (ANOSIM, global *R* = 0.45, p < 0.0019), genital (ANOSIM, global *R* = 0.45, p < 0.0001) and buccal (ANOSIM, global *R* = 0.39, p < 0.0001) secretions between groups (Table 2).

**Table 2:** Differences in odour profiles were found between groups and age categories.

	Genital (n = 109)	Buccal (n = 106)	Temporal (n = 106)
**ANOSIM**			
Group	ANOSIM, global *R* = 0.45, p < 0.0001***	ANOSIM, global *R* = 0.39, p < 0.0001***	ANOSIM, global *R* = 0.45, p < 0.0019**
Age	ANOSIM, global *R* = 0.05 p < 0.16	ANOSIM, global *R* = 0.39, p < 0.0001**	ANOSIM, global *R* = 0.26, p < 0.031*
Sex	ANOSIM, global *R* = 0.14, p < 0.07	ANOSIM, global *R* = 1.21, p < 0.21	ANOSIM, global *R* = 1.27, p < 0.07

### Genotype and overall chemical profile (Partial Mantel test)

To determine whether genetic relatedness is reflected in chemical similarity, the association between Bray–Curtis similarity and pairwise genetic relatedness was tested. No correlation was found for temporal, buccal nor genital secretions and genetic relatedness at the population, group or mother–offspring level (Table 3). However, a weak positive relationship was obtained between relatedness and labial secretions when considering only adult sisters (Mantel’s r = 0.19, n = 14, p = 0.04).

**Table 3:** Correlation between genotype and chemical profile tested at the individual, family group, adult sister and mother–offspring level in African elephants.

	Genital (n = 109)	Buccal (n = 106)	Temporal (n = 106)
**Spearman rank correlation**			
Population	Mantel’s r = 0.006, n = 109, p = 0.4	Mantel’s r = 0.53, n = 106, p = −0.02	Mantel's r = 0.02, n = 106, p = 0.18
Group	Mantel's r = −0.01, n = 30, p = 0.6	Mantel’s r = −0.024, n = 30, p = 0.6	Mantel’s r = −0.024, n = 30, p = 0.63
Adult sister dyads	Mantel’s r = 0.19, n = 14, p = 0.04*	Mantel’s r = 0.10, n = 14, p = 0.16	Mantel’s r = −0.048, n = 14, p = 0.67
Mother–offspring dyads	Mantel’s r = −0.14, n = 18, p = 0.97	Mantel’s r = 0.012, n = 18, p = 0.38	n/a

### Chromatograms and multidimensional scaling (MDS)

**Figure 3:**
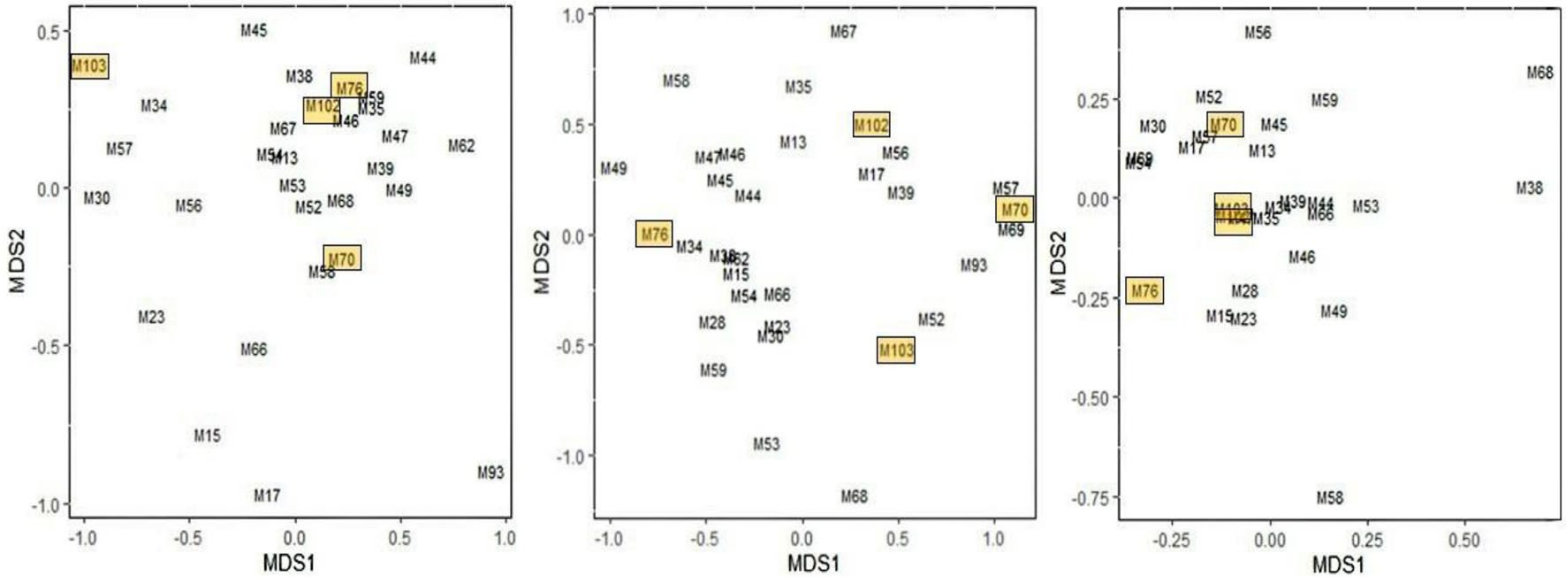
MDS of genital (left), buccal (middle) and temporal (right) secretions in a family core group (Family group L), comprised of four females with high genetic r (QG > 0.45). ♀M**103** (40 years), ♀M**102** (10 years), ♀ M**76** (6 years) ♀M**70** (1 year).

Contrary to our predictions, genetic relatedness was not reflected in temporal gland secretions of adult sisters, nor in buccal, genital and temporal gland secretions of core family units. Highly related sisters and female family members shared similar compounds, which were differentially expressed, as well as individually unique compound (Figures S1 and S2 in Supplementary Information, and Fig. 3).

### Putative identification of important substances

To assess the contribution of specific chemicals to the chemical dissimilarity between groups (Table 4), a “similarity percentages process” (SIMPER) was used. In temporal secretions, 30% of compounds were putatively identified contributing to group dissimilarity (8 compounds) and to chemical dissimilarity between age categories (4 compounds). In genital secretions, eight compounds were identified that contribute to chemical differences among groups, while 15 compounds were identified to contribute towards group differences in buccal secretions.

**Table 4:** Putative compounds and their retention times (RT) identified by Similarity Percentage Analysis (SIMPER) that contributed towards the chemical dissimilarity between elephant groups.

Retention time (RT)	Putative ID
**Temporal**	
15.71	1-Hexene
18.73	Acetic Acid * **
27.59	Pentanoic Acid^*
25.42	**Benzaldehyde**, 4-methoxy-,oxime*^
27.09	Geranyl acetone*^
36.02	Phenol, 2,4-bis(1,1-dimethylethyl)
26.45	Hexanedioic acid, dimethyl ester
27.4	Hexanoic acid* **
**Genital**	
27.54	Pentanoic acid^*
31.87	**Phenol**, 4-methyl-* **^ P
18.73	Acetic acid* **
32.02	**Phenol**, 3-methyl-* ** P
18.64	1-Hexanol, 2-ethyl- * **
25.45	**Hexanoic acid** * **
23.78	Pentanedioic acid, dimethyl ester
36.02	Phenol, 2,4-bis(1,1-dimethylethyl)-
**Buccal**	
27.47	Pentanoic acid^*
25.81	Undecanoic acid, 10-methyl-, methyl ester**
18.61	Acetic acid* **
18.75	Decyl Aldehyde*
18.7	1-Hexanol, 2-ethyl-* **
30.16	**Tetradecanoic acid**, methyl ester* **
25.75	**Dodecanoic acid**, methyl ester* **
15.6	Nonanoic acid, 9-oxo-, methyl ester
34.07	Hexadecanoic acid, methyl ester* **
27.54	Butanoic acid, 3-methyl-^*
34.54	7-Hexadecenoic acid
36.04	Phenol, 2,4-bis(1,1-dimethylethyl)-
18.55	Nonanoic acid, methyl ester**
21.02	**Decanoic acid,** methyl ester* **
23.73	2(3H)-Furanone, 5-ethyldihydro-

Bold semiochemical in elephants^30,32,33,52–57^ **P** Pheromone in elephants. *Confirmed semiochemical in mammals^55^. **of mammalian origin^56^. ^Characterised signaling compound in mammals^57^.

The Similarity Percentage Analysis (SIMPER) revealed that chemical compound categories comprised fatty acids, ketones, aromatic compounds and aldehydes, compounds typically identified in olfactory cues across taxa due to their detectability and volatility^1,2^. Compounds of confirmed mammalian origin were identified in all three body odours (produced by the elephants themselves), and included the saturated fatty acids while others are noted signalling compounds in mammals (Table 4). Chemical compounds in temporal gland secretions identified included short-chain fatty acids (SCFAs), including acetic acid and hexanoic acid, as well as benzaldehyde, geranyl acetone and pentanoic acid. In buccal secretions, the SIMPER analysis identified saturated and short chain fatty acids, comprising acetic acid, tetradecanoic acid, dodecanoic acid, butanoic acid as well as undecanoic acid and pentanoic acid that contributed to the chemical differences among elephant family groups. In genital secretions, SIMPER extracted two phenols (m-and p-cresol) that are known pheromones in elephants.

## Discussion

This study presents the first comprehensive semiochemical analysis of wild African elephants, and investigates whether odour profiles are correlated with genetic relatedness and social relationships. We were specifically interested in the fixed and variable information encoded in temporal gland, buccal and genital odour. Our results demonstrate that cues of identity and maturity may be important components of odour in African elephants. Our analyses also demonstrate the possible mechanism for a group –specific odour in elephants, and which doesn't appear to be a function of genetic relatedness.

The first prediction, that genetic relatedness is correlated with chemical profile at the core (family) unit level, was not supported by the data. Our prediction was based on the apparent importance of genetic relatedness among elephant family units to inclusive fitness benefits, which drives hierarchical structure and underlying elephant sociality^44^. Furthermore, in fission–fusion societies, where females split into multiple subgroups, group-specific odour may facilitate long-term group stability, and such group odour has been confirmed in another fission–fusion mammal—the spotted hyaena^58^ (*Crocuta crocuta)*. Genetic relatedness has been correlated with chemical similarity (known as Odour-Gene Covariance) in a multitude of mammals from primates to rodents^13,23^. The assumption that average genetic relatedness would be high among family groups as females breed within the group into which they are born, was not reflected in the data. Research has subsequently suggested that average kinship amongst related females is low in groups with multiple breeding females from successive generations, and polygynous mating systems, both features that describe elephant society^59^. Further, when the results are viewed and compared with social organization of depredated elephant populations^47^ with low average genetic relatedness, where hierarchical structuring is not genetically based, and non-relatives comprise groups across social tiers, an olfactory group odour based on genetic relatedness seems extraneous. Rather, for sociality to function through the sustained maintenance of cohesive groups, an olfactory signal for social affiliation is more likely to exist in family groups comprised of both related and unrelated females. This is the case in African elephant bulls, where musth odour (dominance rank), secreted by the temporal gland and in urine, stabilizes bull group dominance hierarchies and competitive interactions^28^. The function of the temporal gland secretions of African elephant females remains poorly understood, which is surprising because they secrete it frequently during ritualized greeting ceremonies, while Asian females do not. It is however highly likely that temporal gland secretions regulate social complexity among African elephant females, which display rank derived spatial partitioning and vocalisations^39,48^. We did find that olfactory cues in the labial secretions of adult sisters encoded information about female relatedness. Results are supported by studies on lemurs, where within and between sex genital odour encoded for genetic relatedness in the competitive breeding season^13^. This raises the possibility that African elephant labial odours contain olfactory cues that elicit reciprocal or maternal behaviour among conspecifics of the same family group, which may be particularly relevant in the context of female kin and allomothering. Labial secretions have been shown to generate significant interest in female African elephants^60^. African elephant female elephants establish transitive linear dominance relationships that can determine access to usurpable resources and influence reproductive opportunities^44^. African elephant family groups show defensive responses to unknown, unrelated (less familiar) and less dominant elephants^36^ so genital odour may consequently play an important role in maintaining social dominance.

Our results further indicate olfactory cues for individual identity in buccal, temporal gland and genital odour. Identity cues for individuality in mammalian odour can be expressed by presence/absence of compounds as well as relative proportions of compounds expressed^17^. Visual inspection of the chromatograms confirmed these results. Highly related individuals had their own unique compounds as well as sharing subsets of chemicals that were differentially expressed, within and across age categories and sexes. In support of olfactory individuality cues in African elephants, studies found that females’ and males’ acoustic signals express individuality^61^. The results are supported by research on other group-living mammals, where olfactory cues for individual identity have been documented^3,62^.

Phenotypic variability and distinctiveness in odour profiles might be under natural selection as the ability to identify individuals within a social group may confer fitness benefits by recognizing kin, a heterozygous mate, a social affiliate, a dominant conspecific, or rival, thereby determining agonistic or reciprocal behaviour. The ability to discriminate individual odours has been recorded in a diversity of species^63–68^. African elephants are able to discriminate between humans from body odour and among conspecifics, and prefer the scent of unfamiliar over familiar urine^67^. Females can monitor and track the location of up to 30 conspecifics from odour profiles in urine, while acoustically, they can recall the vocalisations of up to 100 individuals^49,68^.

The last prediction, that age and sex would be encoded in genital, temporal gland and buccal secretions, was supported by the data. Elephant odour was significantly different between age categories in temporal and buccal secretions, while a result approaching significance was obtained for sex in genital and temporal odour. Age class differences are likely due to hormonal variations and varied physical development. Diet, hormonal differences and gut bacteria are known to contribute to odour profiles^69^. Studies suggest that the frequency of African elephant TGS secretion varies with gender and age^70^, while age has been correlated with chemical profile in several mammals^6,7,71,72^. While sex is typically encoded in chemical profiles, here a result approaching significance was detected in genital and temporal secretions, possibly due to the absence of sexually mature males from the sample population as sexual signals pertaining to musth and oestrus are more likely to be encoded in sexually mature adults’ penile, labial and TGS. This would also explain the lack of significance for sex in buccal secretions as mature Asian bulls’ breath and temporal gland secretions are known to contain olfactory signals for musth and reproductive status^33^.

Most intriguingly, while no genetic basis for group odour was found, our results indicated highly significant differences between groups in buccal, genital and temporal gland odour. This raises the possibility that elephants may share a group odour that is not correlated to genetic relatedness. An analysis of similarity elucidated this further, extracting important compounds that contributed towards these differences. Compounds constituted short-chain fatty acids (SCFAs) and saturated fatty acids such as acetic acid, pentanoic acid, hexanoic acid, decanoic acid, dodecanoic acid and tetradecanoic acid (Table S2 in Supplementary Information), compounds that were found by previous studies^24,73^ to be the results of bacterial fermentation and the source of group odour in spotted hyaenas and meerkats (*Suricatta suricata*). Compounds identified in this study were also previously detected in elephant male urine by Goodwin et al. (2016) in a seminal study on microbial communities and production of carboxylic acids. Specifically buccal secretions contained similar compounds, including alkan-2-ones and alkan-2-ols such as octanoic acid, hexanoic acid, decanoic acid, dodecanoic acid and tetradecanoic acid. Notably, the odourants in musth in African elephant bulls derive from bacterial metabolisation of fatty acids. Bacterial activity has been linked to odour profiles in rabbits *Oryctolagus cuniculus*^74^, red foxes (*Vulpes vulpes*) and beavers^75^. Bacteria are proposed to play a significant role in the composition of mammalian odour signals, as found in host urine, faeces or in products from specialized sebaceous or apocrine scent glands; bacteria are suggested to encode information about the sex, age, breeding condition, health, diet, dominance rank and social relationships of their hosts^25^. The fermentation hypothesis for chemical communication^76,77^ suggests that scent glands harbor symbiotic bacteria that decompose organic material and produce volatile compounds, which may contribute to intra-individual recognition in mammals. Evidence supporting the fermentation hypothesis comes from studies on SCFAs in the red fox and mongooses (*Herpestes auropunctatus*). Both species stopped producing SFCAs with the administration of antibiotics, while cultivated bacteria from the scent glands produced the same SCFAs found in scent marks.

Group specific odour is not mutually exclusive to individual specific odour and is suggested to be the result of conspecifics of the same social group harboring more similar bacterial communities than different social groups^78,79^. This was supported by studies on meerkats (*Suricata suricatta*) where scent profiles of anal gland secretions were more similar within than between groups^24^. Studies suggest that cross-infection of bacteria arises through frequent physical contact and allo-or-overmarking within groups ^58,79^. The frequent affiliative behaviours among African elephants is posited as a likely mechanism for bacterial transmission; but also see Chiyo et al. (2014)^80^ for discussion of the role of environment in age-structured transmission of *E.coli* in African elephants.

In summary, this study used SPME-GCMS in conjunction with genetic and statistical tools to test the correlation between genetic relatedness and odour profiles in African elephants. Despite the stress undoubtedly experienced by individuals during translocation, this event provided a unique opportunity to sample at scale the semiochemistry of wild African elephants and to significantly further our understanding of the role of olfaction and chemical cues in social communication. It demonstrated that chemical profiles from temporal gland, buccal and genital secretions coded for individual identity and age. Furthermore, the study showed that group-specific odour in African elephants is not correlated to genetic relatedness, but is likely to be a result of bacteria. Natural selection could favour flexible group-specific bacterial odours that promote bond group cohesion among non-relatives and increase fitness benefits (cooperative offspring care and cooperative group defense) among elephant groups that fluctuate in size and composition over an elephant’s lifetime. African elephant females live in fission–fusion societies, and while family units are stable and long-lasting, across a population, females may change social affiliation at the bond group level several times over their lifetime, requiring olfactory flexibility in advertising group membership. Compounds extracted by an analysis of similarity would be a good starting point for future research into surveying the microbiomes in African elephants using next-generation sequencing methods, and to explore a possible functional role of SFCAs in individual and group odour. Finally, that genetic relatedness was only reflected in chemical similarity of sister dyads in this study does not imply that elephants are unable to recognize kin on the basis of their genetic relationship. Elephants may use other mechanisms to mediate kin recognition including phenotype matching, as well as a possible role for Major Histocompatibility Complex (MHC) derived components or Major Urinary Proteins (MUPs) in elephant urine; all of which remain to be explored in African elephants. Elephants may further use a combination of modalities, such as acoustic and odour-based cues, to recognize conspecifics and kin, and may acquire knowledge of the scent of conspecifics through social learning.

## Methods

### Sample collection

To relieve elephant population pressure in Majete Wildlife Reserve (MWR) in Malawi (S15° 54′26.6″; E034° 44′24.3″), management authorities relocated 120 African elephants in 15 family units to Nkhotakota Wildlife Reserve within Malawi. Translocation of elephants (sighting, selection, immobilization, and transport) and veterinary services were conducted by translocation specialists Conservation Solutions (CS). Elephants travelling as family groups were identified from the air, and darted as a social unit. Permission was granted to Stellenbosch University by The Department of National Parks and Wildlife (DNPW) in Malawi, African Parks (AP) and CS to collect chemical swabs and blood samples under veterinary supervision. Elephants were assigned an individual number, a group membership identification (ID), and were sexed, aged and weighed. Temporal, buccal and genital swabs were taken in triplicate using 3 × sterile COPAN cotton wool swabs, and one swab per individual was used for subsequent analysis. Samples were stored at −20 °C in 20 ml clear precision screw-thread glass vials. Blood was taken from the auricular vein of the elephants by a wildlife veterinarian into 5 × 4 ml sterile EDTA vacutainer tubes. Samples were stored on ice between darting events, and frozen at −20 °C within an hour.

### DNA extraction, microsatellite genotyping and estimating relatedness

Genomic DNA was extracted one month after the translocation using the Prepfiler Automated Forensic DNA extraction kit (Thermo Scientific) and purified on the Kingfisher Flex Purification System (Thermo Scientific). Individuals were genotyped at 18 microsatellite loci using two multiplex panels that comprise previously reported loci^81–83^. For quality control 11 individuals were randomly re-extracted and re-genotyped across the full panel. For each multiplex panel, PCR and electrophoresis were performed on 10 µl reactions using the KAPA2G™ Fast Multiplex PCR Kit (Kapa Biosystems). Amplification PCRs were performed on a GeneAmp PCR System 9800 as follows: 95 °C for 3 min; 30 cycles of 95 °C for 15 s, 60 °C for 15 s and 72 °C for 30 s; and a final amplification at 72 °C for 10 min. Electrophoresis was performed on a 3500 × Genetic Analyzer (Thermo Scientific) and the resulting data were analysed in STRand^84^ using the GeneScan™ 500 LIZ® size standard (Thermo Scientific). To estimate pairwise genetic relatedness between all individuals, the software Identix^85^ was used to calculate Queller and Goodnight’s *r*^86^ for each dyad across the data set. To verify mother–offspring dyads sampled in the field a maternity analysis of cows and calves was performed in Cervus 2.0^87^. Cervus uses a likelihood-based approach to assign parentage combined with simulation of parentage analysis to determine the confidence of parentage assignments.

### Chemical analysis of temporal, buccal and genital samples

Chemical compounds from temporal, buccal and genital secretions were analysed using solid-phase microextraction (SPME) and gas chromatography mass spectrometry (GC–MS). Swabs were transferred into 20 mL SPME headspace vials and sealed with a polytetrafluoroethylene (PTFE, Teflon®)/silicone septa and steel cap. As an internal standard, 50 µL of Anisole d8 was added. Vials were equilibrated at 30 °C for 5 min using a CombiPAL solid-phase microextraction (SPME) autosampler (CTC, Switzerland). A conditioned fibre was inserted into the headspace above the sample and held for 30 min (with agitation). The fibre was consequently withdrawn into the needle by the autosampler and inserted into the injection port of a 6890 N gas chromatograph (GC) (Agilent Technologies, Palo Alto, CA, USA) coupled to a mass spectrometer detector 5975B (Agilent Technologies). The SPME fibre was desorbed and held in the injection port (250 °C) for 10 min. The fiber was then inserted into a fibre conditioning station for 10 min between samples for cleaning to prevent cross-contamination. Volatile compounds were separated using a polar ZB-Wax capillary column (30 m, 0.25 mm i.d. 0.25 µm film thickness). The oven temperature was initially held at 40 °C for 5 min and increased to 240 °C at 5 °C/min (held for 3 min). The total run time was 48 min. Semiochemicals that had consistent retention times and accounted for > 0.05% of the area of the total chromatogram were retained.

### Statistical analysis framework

For statistical analyses of the chemical data, peaks that were present in blanks and only one sample were excluded. To estimate the maximum number of substances within the samples and to test for completeness of sampling, the maximum number of substances using the Michaelis–Menten function with a permutation procedure of 9999 iterations was applied. Analyses were conducted on the relative proportion of each peak in the chromatogram to the overall area of an individual profile. The data were visualized and statistically analysed for patterns of chemical similarity in relation to sample population, social group, adult sister pairs and relatedness. Computer code for R (GCAlign) was used^87^, a package specifically developed for ecological and evolutionary research, evaluating similarity patterns across multiple and variable biological samples.

### Chemical similarity

Chemical profiles were visualized using multidimensional (MDS) scaling ordination and nonmetric multidimensional scaling (nMDS) based on Bray–Curtis Similarity Values calculated from the log(x + 1) transformed data^11,88^. Each point in the 2-dimensional MDS plot represents an individual elephant swab, with clumped points representing individuals with greater chemical similarities. MDS has been successfully used in other studies to visualize chemographic data in mammals^89^. Differences between a priori defined groups (family groups and -sister dyads) were analysed with ANOSIM, using whole profile comparison based on Bray–Curtis indices and applying 999 iterations of the dataset. The vegan package^90^ for ANOSIM was used.

### Partial Mantel tests

Using the full complement of swabs, a correlation of dyadic values of relatedness against chemical similarity was conducted using the genetic relatedness matrix based on 18 loci as the response variable and fitted pairwise Bray–Curtis Similarity Matrices as predictor variables using a Partial Mantel test. Separate models were constructed for a priori defined groups (groups, sister dyads) using 10,000 permutations of the dataset. For the association between relatedness and chemical similarity, Spearman Rank Correlation (Mantel’s r) and two-tailed P-value tests were applied.

### Putative identification of chemicals

To assess the contribution of specific chemicals to the dissimilarity between groups, a similarity percentages process (SIMPER) was used. All Bray–Curtis similarities within a group were decomposed into percentage contributions per compound, listing the compounds in decreasing order of importance.

### Putative identification of compound type

The type of compound represented by each peak was identified by filtering compounds by quality. Compounds were retained that had a probability match of > 70%. Identification of putative substances was based on retention times, and by comparing mass spectra with the best match of the library of the National Institute of Standards and Technology (Gaithersburg, MD, USA). Compounds were categorized into compound categories. Exact identification of each compound, through the injection of commercial standards, was considered unimportant for the present study^62^.

### Ethics statement

All research protocols were approved by Stellenbosch University Institutional Animal Care and Use Committee (SU-ACUM15-00002) and the US Army Medical Research and Materiel Command (USAMRMC) Animal Care and Use Review Office (ACURO) proposal 65978-ST-ITC, and Award W911NF-14-1-0596. All sampling of individuals while sedated was carried out under veterinary supervision. Samples were collected in accordance with the Convention of International Trade in Endangered Species of Fauna and Flora (CITES Permit # Malawi 171383, CITES Permit # South Africa 000054), and retained under permits issued by the Department of Environmental Affairs (DEA Permit # 07901). The translocated elephants analysed in this study are part of an ongoing conservation initiative undertaken through a collaboration between Malawi’s Department of National Parks and Wildlife (DNPW) and the conservation NGO African Parks. The long-term goals of translocation are to maintain healthy habitats in Malawi's national parks, establish stable and resilient elephant populations, and ensure the prosperity of local communities that live alongside them. [See www.africanparks.org]. This study is reported in accordance with ARRIVE guidelines (https://arriveguidelines.org).

## Supplementary Information

Supplementary Information 1.

Supplementary Information 2.

## Data Availability

The datasets generated during and/or analysed during the current study are available from the corresponding author on reasonable request.

## References

[1] Wyatt T (2003). Pheromones and Animal Behavior: Communication by Smell and Taste.

[2] Wyatt TD (2009). Fifty years of pheromones. Nature.

[3] Burgener N, Dehnhard M, Hofer H, East M (2009). Does anal gland scent signal identity in the spotted hyena?. Anim. Behav..

[4] Kent L, Tang-Martínez Z (2014). Evidence of individual odors and individual discrimination in the raccoon, *Procyon lotor*. J. Mamm..

[5] Klücklich M, Weiß BM, Birkemere C, Einspanier A, Widdig A (2019). Chemical cues of female fertility states in a non-human primate. Sci. Rep..

[6] Setchell JM, Vaglio S, Moggi-Cecchi J, Boscaro F, Calamai L, Knapp LA (2010). Chemical composition of scent-gland secretions in an Old World monkey (*Mandrillus sphinx*): Influence of sex, male status, and individual identity. Chem. Sens..

[7] Marneweck C, Jurgens A, Shrader AM (2017). Dung odours signal sex, age, territorial and oestrous state in white rhinos. Proc. R. Soc. B.

[8] Heth G, Todrank J, Busquet N, Baudoin C (2003). Genetic relatedness assessment through individual odour similarities (G-ratios) in mice. Biol. J. Lin. Soc..

[9] Heth G, Todrank J, Begall S, Wegner R, Burda H (2004). Genetic relatedness discrimination in eusocial *Cryptomys anselli* mole-rats, Bathyergidae, Rodentia. Folia Zool..

[10] Busquet N, Baudoin C (2005). Odour similarities as a basis for discriminating degrees of kinship in rodents: Evidence from *Mus spicilegus*. Anim. Behav..

[11] Stoffel MA, Caspers BA, Forcada J, Giannakara A, Baier M, Eberhart-Phillips L, Müller C, Hoffman JI (2015). Chemical fingerprints encode mother–offspring similarity, colony membership, relatedness, and genetic quality in fur seals. PNAS.

[12] Charpentier M, Boulet M, Drea C (2008). Smelling right: The scent of male lemurs advertises genetic quality and relatedness. Mol. Ecol..

[13] Boulet M, Charpentier MJE, Drea CM (2009). Decoding an olfactory mechanism of kin recognition and inbreeding avoidance in primates. BMC Evol. Biol..

[14] Kean EF, Bruford M, Russo IR, Müller C, Chadwick E (2017). Odour dialects among wild mammals. Sci. Rep..

[15] Wedekind C, Seebeck T, Bettens F, Paepke AJ (1995). MHC-dependent mate preferences in humans. Proc. Biol. Sci..

[16] Penn D, Potts WK (1998). Untrained mice discriminate MHC-determined odors. Phys. Behav..

[17] Sun L, Müller-Schwarze D (1998). Anal gland secretion codes for family membership in beaver. Behav. Ecol. Sociobiol..

[18] Bloss J, Acree TE, Bloss JM, Hood WR, Kunz TH (2002). Potential use of chemical cues for colony-mate recognition in the big brown bat, *Eptesicus fuscus*. J. Chem. Ecol..

[19] Weiß BM, Marcillo A, Manser M, Holland R, Birkemeyer C, Widdig A (2018). A non-invasive method for sampling the body odour of mammals. Methods Ecol. Evol..

[20] O'Riain MJ, Jarvis JUM (1997). Colony member recognition and xenophobia in the naked mole-rat. Anim. Behav..

[21] Henkel S, Setchell J (2018). Group and kin recognition via olfactory cues in chimpanzees (*Pan troglodytes*). Proc. R. Soc. B..

[22] Henkel S, Lambides AR, Berger A, Thomsen R, Widdig A (2015). Rhesus macaques (*Macaca mulatta*) recognize group membership via olfactory cues alone. Behav. Ecol. Sociobiol..

[23] Tzur S, Todrank J, Jürgens A, Nevo E, Heth G (2009). Odour–genes covariance within a natural population of subterranean *Spalax galili* blind mole rats. Biol. J. Lin. Soc..

[24] Leclaire S, Jacob S, Greene LK, Dubay GR, Drea CM (2017). Social odours covary with bacterial community in the anal secretions of wild meerkats. Sci. Rep..

[25] Archie E, Theis K (2011). Animal behavior meets microbial ecology. Anim. Behav..

[26] Sukumar R (2003). The Living Elephants: Evolutionary Ecology, Behavior and Conservation.

[27] Jachowski D (2012). The Amboseli Elephants: A long-term perspective on a long-lived mammal by C. J. Moss; H. Croze; P. C. Lee. J. Mammal..

[28] Slotow R, van Dyk G, Poole J, Page B, Klocke A (2000). Older bull elephants control young males. Nature.

[29] Niimura Y, Matsui A, Touhara K (2014). Extreme expansion of the olfactory receptor gene repertoire in African elephants and evolutionary dynamics of orthologous gene groups in 13 placental mammals. Genome Res..

[30] Goodwin TE, Broederdorf LJ, Burkert BA (2012). Chemical signals of elephant musth: Temporal aspects of microbially-mediated modifications. J. Chem. Ecol..

[31] Schulte BA, Rasmussen LEL, Johnston RE, Müller-Schwarze D, Sorensen P (1999). Musth, sexual selection, testosterone and metabolites. Advances in Chemical Communication in Vertebrates.

[32] Rasmussen LEL (1998). Chemical communication: An integral part of functional Asian elephant (*Elephas maximus*) society. Ecoscience.

[33] Rasmussen LEL, Krishnamurthy V (2000). How chemical signals integrate Asian elephant society: The known and the unknown. Zool. Biol..

[34] Greenwood DR, Comesky D, Hunt MB, Rasmussen LEL (2005). Chirality in elephant pheromones. Nature.

[35] Clutton-Brock TH, Huchard E (2013). Social competition and selection in males and females. Phil. Trans. R. Soc..

[36] Wittemyer G, Getz WM (2007). Hierarchical dominance structure and social organization in African elephants *Loxodonta africana*. Anim. Behav..

[37] Moss C (1988). Elephant memories.

[38] Buss IO, Rasmussen LEL, Smuts GL (1976). Role of stress and individual recognition in the function of the African elephants' temporal gland. Mammalia.

[39] Wittemyer G, Douglas-Hamilton I, Getz WM (2005). The socioecology of elephants: Analysis of the processes creating multi-tiered social structures. Anim. Behav..

[40] Bates LA, Sayialel KN, Njiraini NW, Poole JH, Moss CJ, Byrne RW (2008). African elephants have expectations about the locations of out-of-sight family members. Biol. Lett..

[41] Bates LA, Sayialel KN, Njiraini NW, Moss CJ, Poole JH, Byrne RW (2007). Elephants classify human ethnic groups by odor and garment color. Curr. Biol.

[42] Plotnik JM, Brubaker DL, Dale R, Tiller LN, Mumby HS, Clayton NS (2019). Elephants have a nose for quantity. PNAS.

[43] de Silva S, Schmid V, Wittemyer G (2016). Fission–fusion processes weaken dominance networks of female Asian elephants in a productive habitat. Behav. Ecol..

[44] Archie EA, Moss CJ, Alberts SC (2006). The ties that bind: Genetic relatedness predicts the fission and fusion of social groups in wild African elephants. Proc. R. Soc. Lond..

[45] Allen CRB, Brent LJN, Motsentwa T, Weiss MN, Croft DP (2020). Importance of old bulls: Leaders and followers in collective movements of all-male groups in African savannah elephants (*Loxodonta africana*). Sci. Rep..

[46] Goodwin T, Harelimana I, MacDonald L, Mark D, UmuhireJuru A, Yin Q, Engman J, Kopper R, Lichti C, Mackintosh S, Shoemaker J, Sutherland M, Tackett A, Schulte B, Schulte B, Goodwin T, Ferkin M (2016). The Role of Bacteria in Chemical Signals of Elephant Musth. Chemical Signals in Vertebrates.

[47] Wittemyer G, Okello JB, Rasmussen B, Arctander P, Nyakaana S, Douglas-Hamilton I, Siegismund HR (2009). Where sociality and relatedness diverge: The genetic basis for hierarchical social organization in African elephants. Proc. Biol. Sci..

[48] Stoeger A, Baotic A (2016). Information content and acoustic structure of male African elephant social rumbles. Sci. Rep..

[49] McComb K, Reby D, Baker L, Moss C, Sayialel S (2003). Long-distance communication of social identity in African elephants. Anim. Behav..

[50] Archie EA, Hollister-Smith JA, Poole JH, Lee PC, Moss CJ, Maldonado JE, Fleischer RC, Alberts SC (2007). Behavioural inbreeding avoidance in wild African elephants. Molec. Ecol.

[51] von Dürckheim, K. *Olfaction and scent discrimination in African elephants*. PhD thesis, Stellenbosch University, South Africa (2021).

[52] Goodwin TE, Brown FD, Counts RW, Dowdy NC, Fraley PL, Hughes RA, Liu DZ, Mashburn CD, Rankin JD, Roberson RS, Wooley KD, Rasmussen LEL, Riddle SW, Riddle HS, Schulz S (2002). African elephant sesquiterpenes. II. Identification and synthesis of new derivatives of 2,3-dihydrofarnesol. J. Nat. Prod..

[53] Goodwin TE, Rasmussen LEL, Schulte BA, Brown PA, Davis BL, Dill WM, Dowdy NC, Hicks AR, Morshedi RG, Mwanza D, Loizi H, Mason RT, LeMaster MP, Müller-Schwarze D (2005). Chemical analysis of African elephant urine: A search for putative pheromones. Chemical Signals in Vertebrates 10.

[54] Goodwin TE, Eggert MS, House SJ, Weddell ME, Schulte BA, Rasmussen LEL (2006). Insect pheromones and precursors in female African elephant urine. J. Chem. Ecol..

[55] Burger BV, Schulz S (2005). Mammalian semiochemicals. The chemistry of Pheromones and Other Semiochemicals II. Topics in Current Chemistry.

[56] Charpentier MJE, Barthes N, Proffit M, Bessière JM, Grison C (2012). Critical thinking in the chemical ecology of mammalian communication: Roadmap for future studies. Funct. Ecol..

[57] Apps P, Weldon P, Kramer M (2015). Chemical signals in terrestrial vertebrates: Search for design features. Nat. Prod. Rep..

[58] Burgener N, East M, Hofer H, Dehnhard M, Hurst JL, Beynon RJ, Roberts SC, Wyatt TD (2008). Do spotted hyena scent marks code for clan membership?. Chemical Signals in Vertebrates XI.

[59] Lukas D, Clutton-Brock T (2018). Social complexity and kinship in animal societies. Ecol. Lett..

[60] Meyer JM, Goodwin TE, Schulte BA (2008). Intrasexual chemical communication and social responses of captive female African elephants, *Loxodonta africana*. Anim. Behav..

[61] Soltis J, Leong K, Savage A (2005). African elephant vocal communication II: Rumble variation reflects the individual identity and emotional state of callers. Anim. Behav..

[62] Scordato ES, Drea CM (2007). Scents and sensibility: Information content of olfactory signals in the ringtailed lemur, *Lemur catta*. Anim. Behav..

[63] Palagi E, Dapporto L (2006). Beyond odor discrimination: Demonstrating individual recognition by scent in *Lemur catta*. Chem. Sens..

[64] Johnston RE, Derzie A, Chiang G, Jernigan P, Lee HC (1993). Individual scent signatures in golden hamsters: Evidence for specialization of function. Anim. Behav..

[65] Coffin H, Watters J, Mateo J (2011). Odor-based recognition of familiar and related conspecifics: A first test conducted on captive Humboldt Penguins (*Spheniscus humboldti*). PLoS ONE.

[66] Leclaire S, Merkling T, Delgado Raynaud C, Giacinti G, Bessière J, Hatch S, Danchin E (2011). An individual and a sex odor signature in kittiwakes? Study of the semiochemical composition of preen secretion and preen down feathers. Naturwissenschaften.

[67] von Dürckheim K, Hoffman LC, Leslie A, Hensman MC, Hensman S, Schultz K, Lee S (2018). African elephants (*Loxodonta africana)* display remarkable olfactory acuity in human scent matching to sample performance. Appl. Anim. Behav..

[68] Bates LA, Poole JH, Byrne RW (2008). Elephant cognition. Curr. Biol..

[69] Kean E, Müller C, Chadwick E (2011). Otter scent signals age, sex, and reproductive status. Chem. Sens..

[70] Kioko J, Taylor K, Milne HJ, Hayes KZ, Kiffner C (2017). Temporal gland secretion in African elephants (*Loxodonta africana*). Mamm. Biol..

[71] Macdonald E, Fernandez-Duque E, Sian E, Hagey L (2008). Sex, age, and family differences in the chemical composition of owl monkey (*Aotus nancymaae*) subcaudal scent secretions. Am. J. Primatol..

[72] Zhang J, Liu D, Sun L, Wei R, Zhang G, Wu H, Zhang H, Zhao C (2008). Potential chemosignals in the anogenital gland secretion of giant pandas, *Ailuropoda melanoleuca*, associated with sex and individual identity. J. Chem. Ecol..

[73] Theis KR, Venkataraman A, Dycus JA, Koonter KD, Schmitt-Matzen EN, Wagner AP, Holekamp KE, Schmidt TM (2013). Symbiotic bacteria appear to mediate hyena social odors. Proc. Natl. Acad. Sci..

[74] Merritt GC, Goodrich BS, Hesterman ER, Myktowycz R (1982). Microflora and volatile fatty acids present in the inguinal pouches of the wild rabbit, *Oryctolagus cuniculus* in Australia. J. Chem. Ecol..

[75] Müller-Schwarze D, Heckman S (1980). The social role of scent in beaver (*Castor canadensis*). J. Chem. Ecol..

[76] Albone ES, Eglinton G, Walker JM, Ware GC (1974). Anal sac secretion of red fox (*Vulpes vulpes*), its chemistry and microbiology: Comparison with anal sac secretion of lion (*Panthera leo*). Life Sci..

[77] Gorman ML (1976). A mechanism for individual recognition by odour in *Herpestes auropunctatus* (Carnivora: Viverridae). Anim. Behav..

[78] Theis KR, Schmidt MS, Holekamp KE (2012). Evidence for a bacterial mechanism for group-specific social odors among hyenas. Sci. Rep..

[79] Theis KR, Heckla AL, Verge JR, Holekamp KE, Hurst JL, Beynon RJ, Roberts SC, Wyatt TD (2008). The ontogeny of pasting behavior in free-living spotted hyenas, *Crocuta crocuta*. Chemical Signals in Vertebrates.

[80] Chiyo PI, Grieneisen LE, Wittemyer G, Moss CJ, Lee PC, Douglas-Hamilton I, Archie EA (2014). The influence of social structure, habitat, and host traits on the transmission of Escherichia coli in wild elephants. PLoS ONE.

[81] Archie EA, Moss CJ, Alberts SC (2003). Characterization of tetranucleotide microsatellite loci in the African Savannah elephant (*Loxodonta africana africana*). Mol. Ecol. Notes..

[82] Comstock KE, Wasser SK, Ostrander EA (2000). Polymorphic microsatellite DNA loci identified in the African elephant (*Loxodonta africana)*. Mol. Ecol..

[83] Eggert LS, Eggert JA, Woodruff DS (2003). Estimating population sizes for elusive animals: The forest elephants of Kakum National Park, Ghana. Mol. Ecol..

[84] Toonen RJ, Hughes S (2001). Increased throughput for fragment analysis on an ABI PRISM 377 automated sequencer using a membrane comb and STRand software. Biotechniques.

[85] Belkhir K, Castric V, Bonhomme F (2002). IDENTIX, a software to test for relatedness in a population using permutation methods. Mol. Ecol. Notes.

[86] Queller D, Goodnight K (1989). Estimating relatedness using genetic markers. Evolution.

[87] Marshall TC, Slate J, Kruuk LEB, Pemberton JM (1998). Statistical confidence for likelihood-based paternity inference in natural populations. Mol. Ecol..

[88] Ottensmann M, Stoffel MA, Nichols HJ, Hoffman JI (2018). GCalignR: An R Package for aligning gas-chromatography data for ecological and evolutionary studies. PLoS ONE.

[89] Morelli T, Hayes R, Nahrung H, Goodwin TE, Harelimana I, Macdonald L, Wright P (2013). Relatedness communicated in lemur scent. Naturwissenschaften.

[90] Oksanen, J., Blanchet, F., Guillaume. F., Kindt, R., Legendre, P., Minchin, P., O’Hara, R.B., Simpson, G., Solymos, P., Stevens, M.H.H., Wagner, H. Vegan: community ecology package. R package vegan, vers. 2.2-1. (2015).

