# Supplementary material for: A pachyderm perfume: odour encodes identity and group membership in African elephants
Source: Sci Rep. 2022 Oct 6;12:16768. doi: 10.1038/s41598-022-20920-2 (PMC9537315; doi:10.1038/s41598-022-20920-2)
Supplement: Supplementary file 2 — Supplementary Information 2. [file 41598_2022_20920_MOESM2_ESM.docx]

**
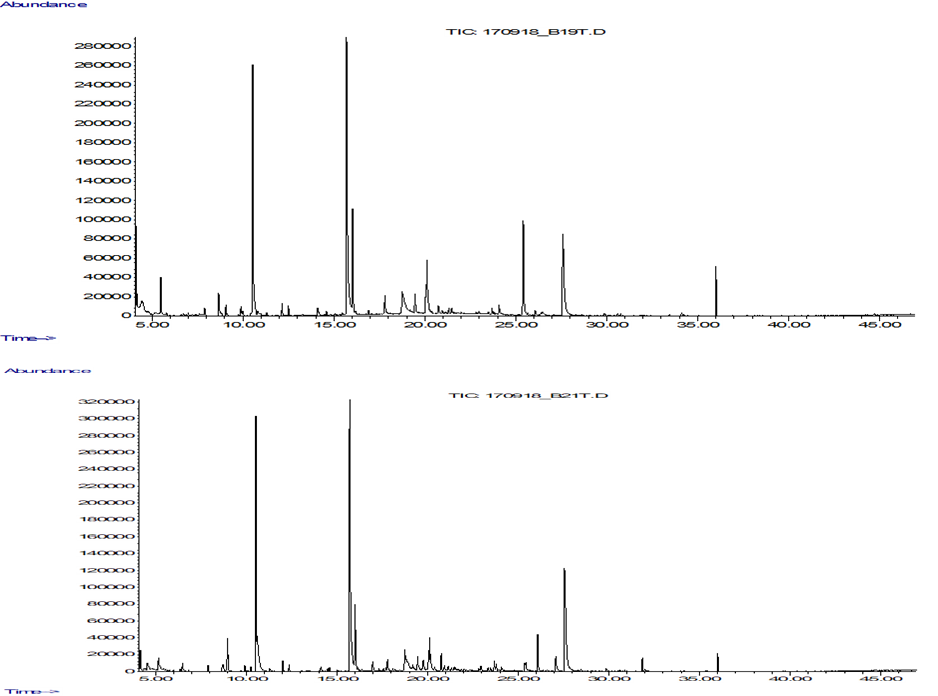
**

**Figure S1:** Total ion chromatogram of temporal gland secretions for two highly related adult sisters ♀ 19 (top) and ♀21 (bottom) with QG>0.6.

**Figure S2:** Total ion chromatogram of genital (column 1), buccal (column 2) and temporal (column 3) secretions in family core group (Herd L), comprised of related females, with high genetic r (QG >0.45). ♀103 (row 1) 40 years, ♀102 (row 2) 10 years, ♀76 (row 3) 6 years, ♀70 (row 4) 1 year old.


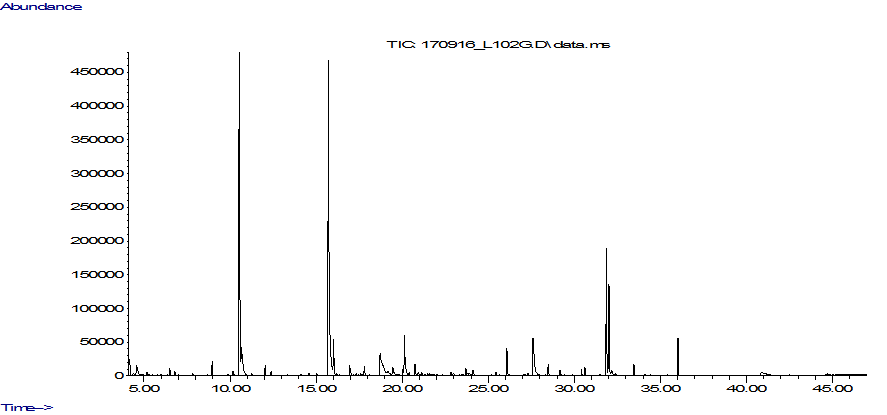

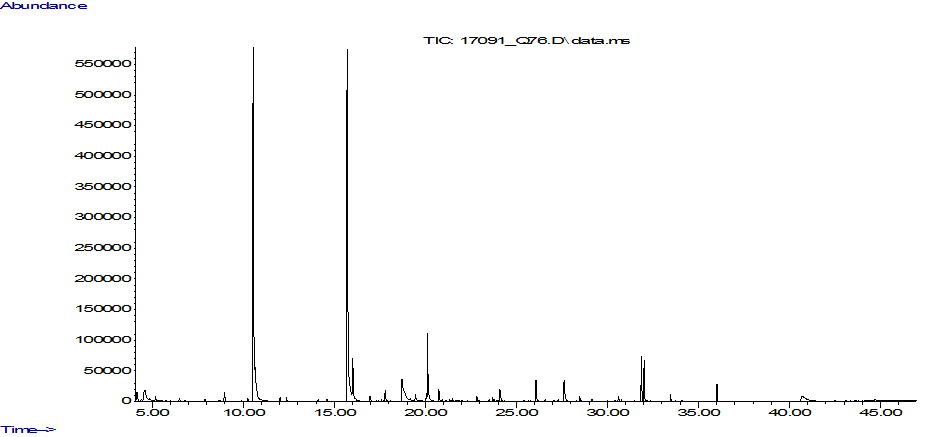

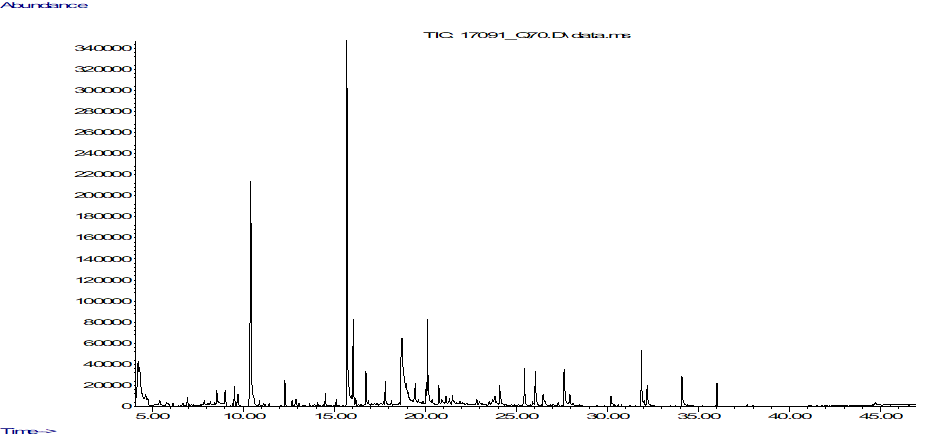

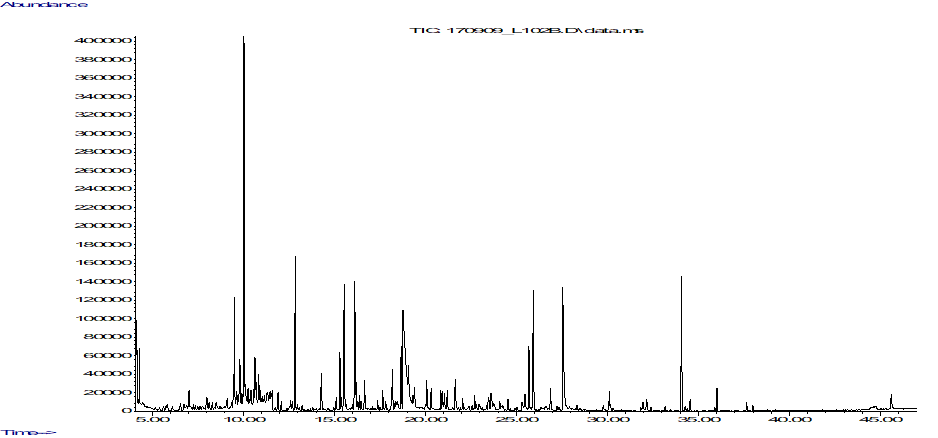

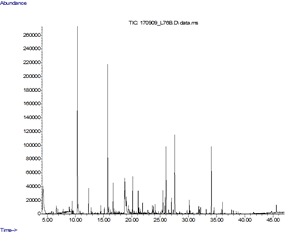

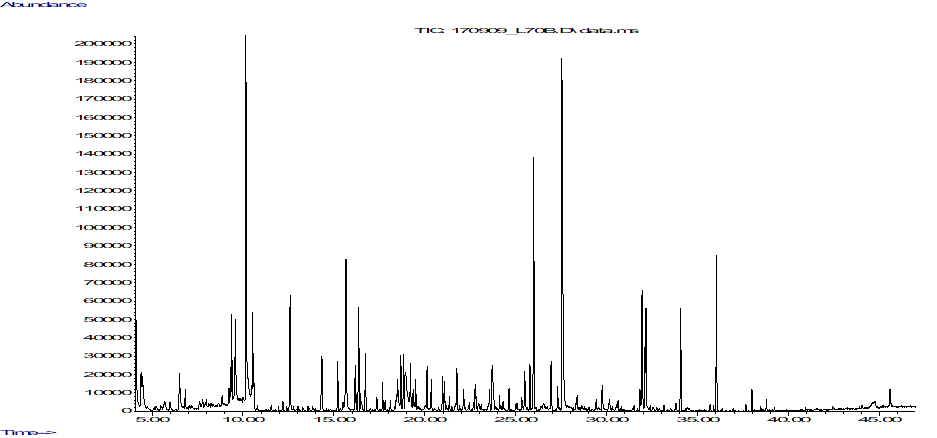

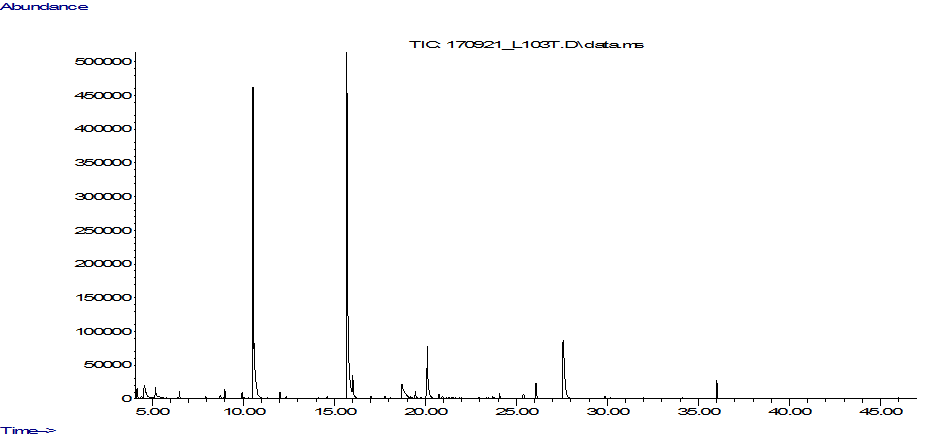

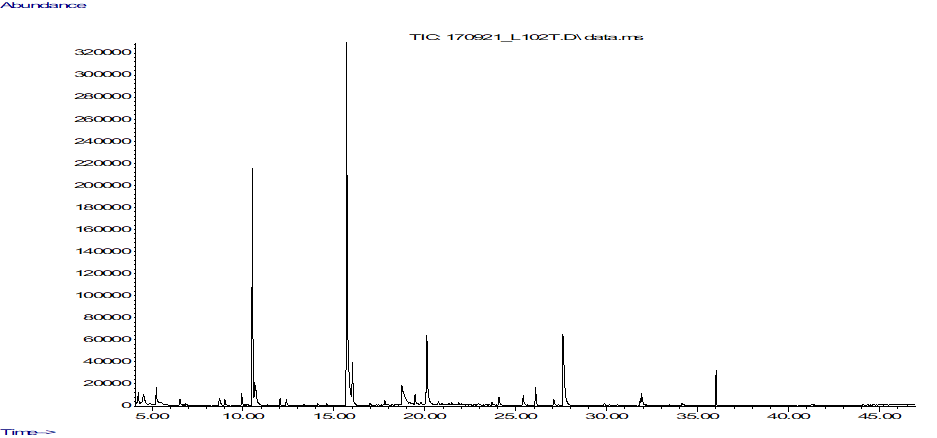

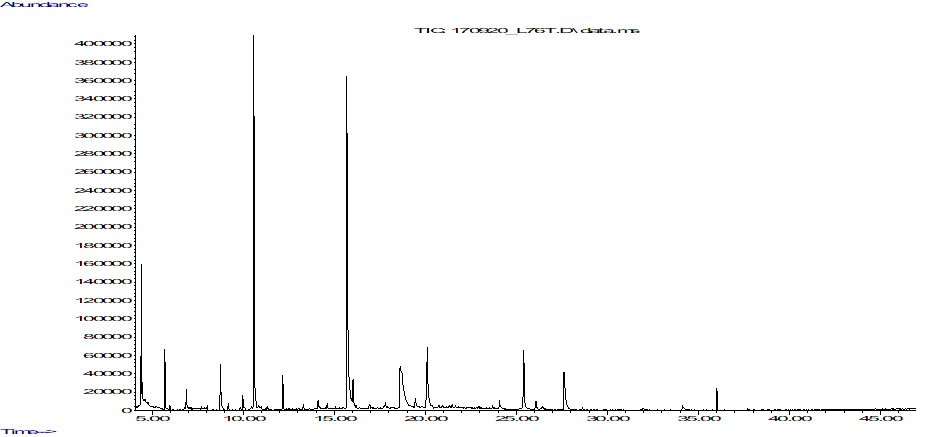

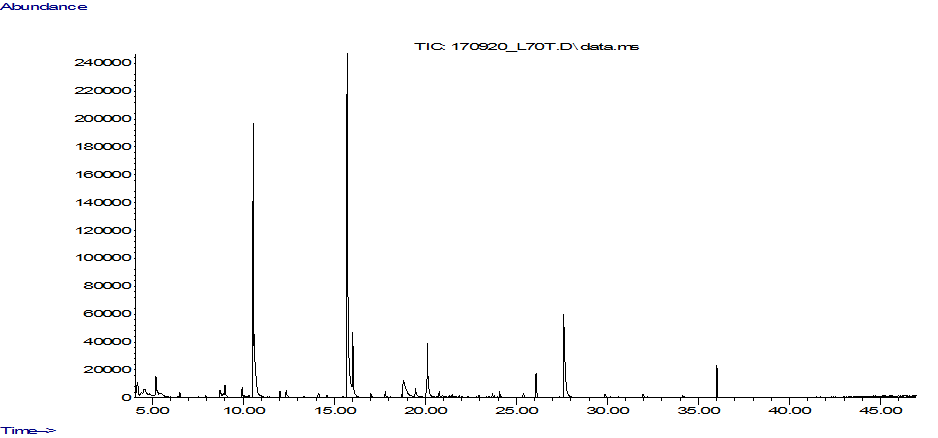

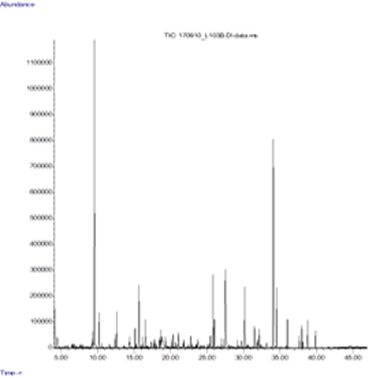

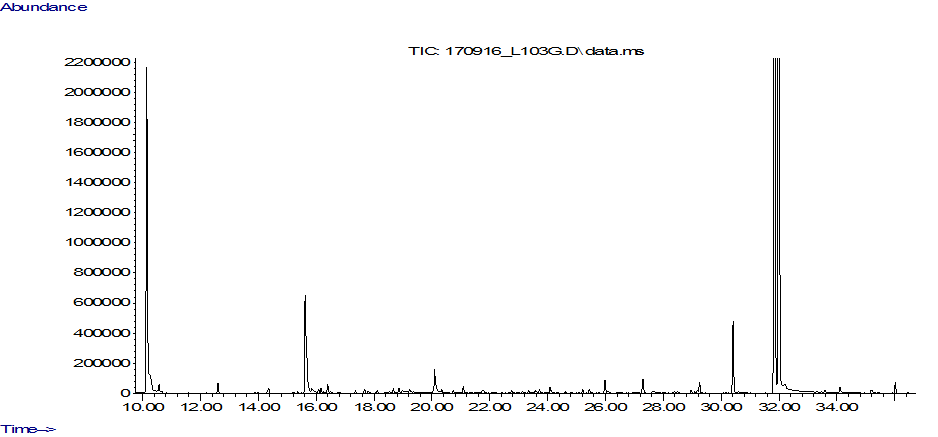


**Table S2:** Volatile Short Chain Fatty Acids (SFCAs) and Low Molecular Weight (LMW) compounds produced by bacteria in hyaenas and meerkats were also found in African elephant scent marks and body odour (von Dürckheim 2021). x* - females only, X** males only, Simper (n= 113), NIST n=40 (adult females only)

|  | **TGS** |  | **Buccal** |  | **Genital** |  | **Urine** |  |
| --- | --- | --- | --- | --- | --- | --- | --- | --- |
|  | SIMPER | NIST | SIMPER | NIST | SIMPER | NIST | SIMPER | NIST |
| **SCFAs in hyenas**  (after Theis et al. 2013) |  |  |  |  |  |  |  |  |
| Acetic/Ethanoic acid | x | x | x | x | x | x |  |  |
| Propionic acid |  |  |  |  |  |  |  |  |
| Butyric/Butanoic acid |  | x | x | x |  |  |  | x |
| Isobutyric/ 2-methylpropanoic acid |  |  |  |  |  |  |  | x* |
| Valeric/Pentanoic acid | x | x | x | x | x | x |  | x |
| Isovaleric acid |  |  |  |  |  |  |  |  |
| Caproic/Hexanoic acid | x | x |  | x |  | x |  | x** |
| **LMW compounds in meerkats**  **(**after Leclaire et al. 2017) |  |  |  |  |  |  |  |  |
| 1-hexadecanol |  |  |  |  |  |  |  |  |
| Lauric/Dodecanoic acid |  |  |  | x |  | x |  |  |
| 1-dodecene |  |  |  | x |  |  |  |  |
| Myristic/Tertradecanoic acid |  |  |  | x |  |  |  |  |
| **Fatty Acids in elephant male urine (**after Goodwin et al. 2016**)** |  |  |  |  |  |  |  |  |
| Caproic/Hexanoic acid | x |  |  | x | x | x |  | x |
| Octanoic/Caprilic acid |  |  |  | x |  |  |  | x |
| Capric/Decanoic acid |  |  | x | x |  |  |  | x |
| Lauric/Dodecanoic acid |  |  | x | x |  | x |  |  |
| Myristic/Tetradecanoic acid |  |  | x | x |  | x |  |  |
